# Advances in the management of acute cholecystitis

**DOI:** 10.1002/ags3.12240

**Published:** 2019-02-19

**Authors:** Danny Mou, Tomas Tesfasilassie, Sameer Hirji, Stanley W. Ashley

**Affiliations:** ^1^ Harvard Medical School CRICO Scholar in Quality and Safety Brigham and Women's Hospital Boston Massachusetts; ^2^ Department of Surgery Harvard Medical School Brigham and Women’s Hospital Boston Massachusetts; ^3^ School of Medicine University of California San Francisco California; ^4^ Brigham and Women's Hospital Boston Massachusetts

**Keywords:** cholecystectomy, cholecystitis, cholelithiasis, laparoscopic cholecystectomy, percutaneous cholecystostomy

## Abstract

The diagnosis and management of acute cholecystitis (AC) continues to evolve. Among the most common surgically treated conditions in the USA, appropriate diagnosis and management of AC require astute clinical judgment and operative skill. Useful diagnostic and grading systems have been developed, most notably the Tokyo guidelines, but some recent clinical validation studies have questioned their generalizability to the US population. The timing of surgical intervention is another area that requires further investigation. US surgeons traditionally pursue laparoscopic cholecystectomy (LC) for AC patients with symptoms onset <72 hours, but for patients with symptoms over 72 hours, surgeons often elect to treat the patients with antibiotics and delay LC for 4‐6 weeks to permit the inflammation to subside. This practice has recently been called into question, as there are data suggesting that LC even for AC patients with over 72 hours of symptoms confers decreased morbidity, shorter length of stay, and reduced overall healthcare costs. Finally, the role of percutaneous cholecystostomy (PC) needs to be better defined. Traditional role of PC is a temporizing measure for patients who are poor surgical candidates. However, there are data suggesting that in AC patients with organ failure, PC patients suffered higher mortality and readmission rates when compared with a propensity‐matched LC cohort. Beyond diagnosis, the surgical management of AC can be remarkably challenging. All surgeons need to be familiar with best‐practice surgical techniques, adjunct intra‐operative imaging, and bail‐out options when performing LC.

## 1 INTRODUCTION

The prevalence of gallstones is reported to range between 10% and 15% among adults, making it one of the most common gastroenterological conditions.^1^ In Western societies, cholesterol gallstones account for 80%‐90% of the stones analyzed after cholecystectomy. Approximately 80% of gallstones remain asymptomatic.^2^, ^3^ Gallstones can obstruct the cystic duct, which can cause gallbladder (GB) distension and biliary colic. Prolonged obstruction results in inflammation, infection, and even ischemia, a common condition known as acute cholecystitis (AC). Approximately 1%‐2% of individuals with gallstones become symptomatic each year.^2^, ^4^ Of those with symptomatic gallstones, 10% will develop AC.^5^ In people under 50 years of age, women are three times more likely than men to develop AC.^6^ Repeated episodes of AC can result in chronic cholecystitis, a condition characterized by thickened GB wall, GB mucosal atrophy, and scarring.

Laparoscopic cholecystectomy (LC) is the standard treatment for AC. LC has replaced open cholecystectomy (OC) as the first‐line treatment for AC, as it confers comparable effectiveness, lower morbidity, and lower costs.^7^, ^8^ An analysis of the National Hospital Discharge Survey (NHDS) from 2000 to 2005 revealed that compared to OC, LC resulted in an increased likelihood of same‐day discharge from the hospital (91% vs 70%), reduced morbidity (16% vs 36%), and lower unadjusted mortality (0.4% vs 3%).^9^ Furthermore, the conversion rate from LC to OC was 9.5%. Interestingly, LC cases that were converted to OC still had lower morbidity and mortality than cases that were initiated as OC, suggesting that early LC should be the treatment of choice for AC.

Not all results are consistent with this data. A 2‐year prospective multicenter survey of over 1000 patients in Belgium, including all centers, revealed that LC and OC approaches were employed in 93.2% and 6.8% of patients, respectively.^10^ Independent predictive factors of an initial OC approach included history of upper abdominal surgery, age over 70 years, surgeons with more than 10 years of experience, and gangrenous cholecystitis. The conversion rate from LC to OC was 11.4%. Bile duct injuries, a devastating complication, occurred in 2.7% of the OC group and 1.1% of the LC group.^10^ However, in those patients whose operation was started laparoscopically but who were converted to open, 13.7% suffered some form of biliary complication. These results suggest that operation for AC can still be associated with a significant complication rate and that we need to continue to evaluate our approach to the difficult cholecystectomy. There continue to be several areas of significant controversy (Table 1).

**Table 1:** Controversial areas of AC diagnosis and management that require further investigation

Clinically relevant AC severity grading	Although the Tokyo Guidelines have provided a severity grading system, its applicability in US populations have been questioned.^18^, ^19^
Timing of surgical intervention	AC patients with over 72 h of symptoms may benefit from early LC as opposed to delayed LC in 4‐6 wks.^30^
Indications for PC	In grade III AC patients, a propensity‐matched cohort study showed that those who received PC and interval LC did worse than those who did not receive PC.^19^
Optimal approach to the difficult cholecystectomy	There continue to be significant differences in the approach to the difficult cholecystectomy, including the failure of surgeons to identify the critical view.^44^

AC, acute cholecystitis; LC, laparoscopic cholecystectomy; PC, percutaneous cholecystostomy

## 2 DIAGNOSIS AND CLASSIFICATION

Accurate diagnosis of cholecystitis requires a multifactorial, systematic approach that involves a detailed history, physical exam, serologic tests, and imaging. The 2007 Tokyo Guideline (TG07) provided a system of diagnostic criteria and severity grading scale for cholecystitis.^11^ Subsequent studies revealed that the TG07 guidelines had a sensitivity and specificity of 85% and 50%, respectively.^12^ The suboptimal specificity prompted a revision of the TG07 to include local and systemic signs of inflammation, as well as imaging findings. These new diagnostic criteria resulted in the 2013 Tokyo Guidelines (TG13), with improved sensitivity and specificity of 91% and 97%, respectively.^13^ Since the establishment of TG13, a review of 216 articles, including 19 randomized controlled trials, showed that the severity grading accurately predicted mortality,^14^ length of hospitalization, and laparotomy conversion rates.^15^, ^16^ Given these findings, the decision was made to decline further revisions to TG13 in the updated Tokyo Guideline 2018 (TG18).

The TG18 diagnostic criteria for AC include three components: (A) local signs of inflammation (e.g., Murphy's sign or right upper quadrant [RUQ] mass/pain/tenderness); (B) systemic signs of inflammation (e.g., fever, elevated C‐reactive protein, elevated white blood cell [WBC] count); and (C) imaging findings. Presence of a finding in the A category and a finding in the B category constitutes a suspected diagnosis, whereas presence of a finding in the A, B, and C categories constitutes a definitive diagnosis.^16^

For the severity grading of cholecystitis, the TG18 preserved the TG13 recommendations. Grade I AC is defined as AC in a healthy patient without organ dysfunction but with evidence of mild inflammation. Grade II is defined as AC with elevated WBC count over 18 000 cells/mm^3^, palpable tender mass at RUQ, symptoms lasting >72 hours, or evidence of marked local inflammation (e.g., gangrenous cholecystitis, pericholecystic abscess, hepatic abscess, biliary peritonitis, or emphysematous cholecystitis).^17^ Grade III AC is characterized by AC with associated organ dysfunction (e.g., hypotension requiring vasopressors, decreased level of consciousness).

Although the TG18 criteria have been validated in Japan, their clinical utility has been questioned in the USA. For example, one recent study from the University of Arizona analyzed a 3‐year prospective database of 857 patients with suspected AC. Of the patients with severe local inflammation, including gangrenous cholecystitis, 45% of them did not meet the TG criteria for diagnosis. The TG sensitivity within this study was only 53%.^18^ The lack of sensitivity has been attributed to the fact that many of the patients with early AC do not exhibit fevers or leukocytosis. The most sensitive AC findings were right upper quadrant abdominal pain and Murphy's sign.^18^ A potential explanation for the different validation results in Japan and in the USA may be that Americans have a lower threshold to seek medical treatment for abdominal pain. The authors concluded that TG13 recommendations for grade II and grade III AC may not necessarily apply to the US population.

Another objection to the Tokyo Guidelines arose from a study in Texas. For grade III AC patients, the 2013 Tokyo Guidelines recommend initial percutaneous cholecystostomy (PC), antibiotics, and delayed cholecystectomy. However, using Medicare data from 1996 to 2010, researchers compared grade III AC patients who received PC to those who did not, and showed that the PC group had higher 30‐day, 90‐day, and 2‐year mortality, increased readmissions, and lower probability of undergoing cholecystectomy within 2 years of hospital admission in older patients with grade III cholecystitis.^19^ In light of these findings, the authors suggested the need for further critical evaluation and possible refinement of the Tokyo Guidelines. The Tokyo Guidelines 2018 has since been revised to recommend that grade III AC can be effectively managed with early LC at advanced institutions with specialized surgeons.^20^

## 3 TIMING OF SURGICAL INTERVENTION

The relationship between AC outcomes and the timing of surgical intervention has been the subject of ongoing study. Initial studies concluded that early LC for AC was associated with a higher conversion rate, more complications, and longer surgery times.^21^ However, with advances in laparoscopic techniques, early LC became the standard practice for treatment of AC. Comparisons between early LC and early OC for AC revealed no significant differences in procedure time, morbidity, or mortality. Furthermore, LC had a significantly shorter postoperative recovery time.^22^, ^23^

Subsequent studies, including several meta‐analyses, suggested that it was appropriate to pursue LC should an AC patient present within 72 hours of symptom onset. The data suggest that early LC leads to superior outcomes. A meta‐analysis of 77 case‐control studies revealed a statistically significant decrease in mortality, complications, bile leakage, wound infections, and conversion for LC done <72 hours compared to LC done between 72 hours and 4 weeks after admission.^24^ An analysis of the Nationwide Inpatient Sample from 2005 through 2009 further investigated operation timing by subdividing LC patients into three groups based on the number of days from admission to LC: 0‐1 day, 2‐5 days, and 6‐10 days. Compared to LC on day 0‐1, patients with LC on days 2‐5 and 6‐10 had increased hospital costs and higher odds of mortality at 1.26 (95% CI, 1.00‐1.58) and 1.93 (95% CI, 1.38‐2.68), respectively.^25^ LC on days 6‐10 also had higher odds of postoperative infection at 1.53 (95% CI, 1.05‐2.23). There was no difference in hospital stay.^25^ Based on these data, it has become more or less the standard of care that, if the patient has had symptoms for >72 hours, clinicians will “cool down” the patient with antibiotics and pursue delayed LC in 4‐6 weeks.

More recent studies have questioned this approach. Specifically, the time course of surgical intervention for AC has been re‐evaluated, including the efficacy of early LC compared to more delayed LC. When comparing an early LC (<72 hours after hospital admission) group to a delayed LC (>6 weeks after hospital admission) group after conservative treatment with antibiotics, the early LC cohort had a significantly reduced operation time and hospital stay.^26^ Conversion rate to open surgery and overall complication rates did not differ significantly between the two groups.^26^, ^27^ Likewise, it has been shown that of those patients with AC who are managed non‐surgically, 9.7%‐23% fail treatment and undergo emergency LC,^27^, ^28^ which is associated with significantly higher mortality, morbidity, and conversion rate than elective LC.^29^ At least one recent trial randomizing patients to less than or more than 72 hours between onset of symptoms and LC found no significant differences in outcomes.^30^ We need further studies comparing delayed LC (>72 hours) to intentional “cool down” with operation in 4‐6 weeks.

In summary, if possible, LC should be performed within 72 hours of presentation for patients with AC. In fact, the Tokyo Guidelines from 2013 and 2018 have recommended LC be performed soon after admission and within 72 hours from onset of symptoms.^20^, ^31^ For patients with symptom onset beyond 72 hours, recent studies suggest that patients still have better outcomes with earlier LC as compared to delayed LC at 4‐6 weeks. Further investigation is warranted to strengthen this finding.

## 4 THE DIFFICULT CHOLECYSTECTOMY

It would be remiss to not include a brief overview of the evolving surgical techniques in addressing the difficult cholecystectomy which most commonly is associated with AC. Patients with technically difficult cholecystectomies are at significantly higher risk for conversion to OC and are at higher risk for biliary duct injury (BDI).^10^ Risk factors that predict a difficult operation include symptoms >72 hours, WBC count greater than 18 000/mm^3^, a palpable GB, or a gangrenous GB. Despite these predictors, LC may prove difficult and not exhibit any of these characteristics.^18^

Protocols have been developed to address the difficult cholecystectomy. The Society of American Gastrointestinal and Endoscopic Surgeons (SAGES) has created a six‐step Safe Cholecystectomy Program.^32^ The principles include the following: (a) achieving the critical view of safety (CVS); (b) recognizing aberrant anatomy; (c) performing an intra‐operative time‐out before clipping or cutting ductal structures; (d) liberal use of intraoperative cholangiogram (IOC); (e) devising bail‐out options; and (f) asking for help in difficult cases. Achieving the CVS is defined by three criteria: (a) the hepatocystic triangle is cleared of fat and fibrous tissue; (b) the lower one‐third of the GB is separated from the liver to expose the cystic plate; and (c) no more than two structures should be seen entering the GB.^33^ Failure to achieve CVS increases the risk of BDI. Despite these recommendations, there exists confusion among surgeons regarding what constitutes the CVS. A Netherlands study reviewed surgical videos of cases with complications and showed that although operative notes indicated that the CVS was achieved in 80%, the video review suggested that it was achieved in only 10.8%.^34^

Should the inflammation be so significant that further dissection is deemed too risky, an IOC can be used to define the biliary anatomy. Some novel technologies such as infrared fluorescent cholangiography have been employed and may be validated.^35^ If these techniques fail to provide sufficient anatomical guidance, conversion to an open procedure is likely indicated. However, before conversion, thoughtful judgement is needed to determine whether an open approach will significantly facilitate the dissection.

All surgeons need to have bail‐out options in their surgical armamentarium when the CVS cannot be achieved. Resecting the GB from the “dome down” is an option, although it is not without significant risk. If only the dome of the GB is exposed, operative cholecystostomy tube placement may be appropriate. If the hepatocystic triangle cannot be safely defined, the surgeon may perform a subtotal cholecystectomy, leaving the posterior wall on the liver.^36^ Usually, a minimum of 2 cm GB neck is preserved, and impacted stones are removed. The neck can be either left open (fenestrating) or oversewn (reconstituting), and a drain is left in the GB fossa.

## 5 PERCUTANEOUS CHOLECYSTOSTOMY

Postoperative mortality rates in LC for high‐risk patients such as the elderly or critically ill have been estimated at 5%‐30%.^37^ Among these patients, PC has been a preferred alternative as this procedure decreases postoperative mortality rates in high‐risk patients to 10%‐12%.^37^ It is important to note that PC can be a technically difficult procedure with potentially high conversion rates. In one study, PC within 2 days of symptom onset had an 8.3% conversion rate, whereas PC between 3 and 6 days from symptoms onset had a 33.3% conversion rate.^38^ Similarly, PC done within 2 days of admission vs 3‐6 days of admission had conversion rates of 16% and 40.7%, respectively.^38^

A 10‐year study at the Veterans Affairs Boston Healthcare System revealed that compared to cholecystectomy (open and laparoscopic), PC was associated with significantly longer intensive care unit (ICU) stays, longer hospital stays, more complications, and higher readmission rates.^39^ The PC and surgical cohorts had comparable mean body temperatures, time of diagnosis after symptom onset, time of antibiotic initiation, and AC severity grade. However, the PC patients were older, with higher WBC counts, alkaline phosphatase levels, Charlson comorbidity index scores, and American Society of Anesthesiologists classes compared to the cholecystectomy group.^39^ These factors likely contributed to the worse outcomes in the PC group. When ICU patients with similar preoperative characteristics underwent PC or emergency cholecystectomy (EC), the EC cohort had a slightly higher mortality rate and a significantly higher morbidity rate (8.7% after PC and 47% after EC).^40^

**Figure 1:**
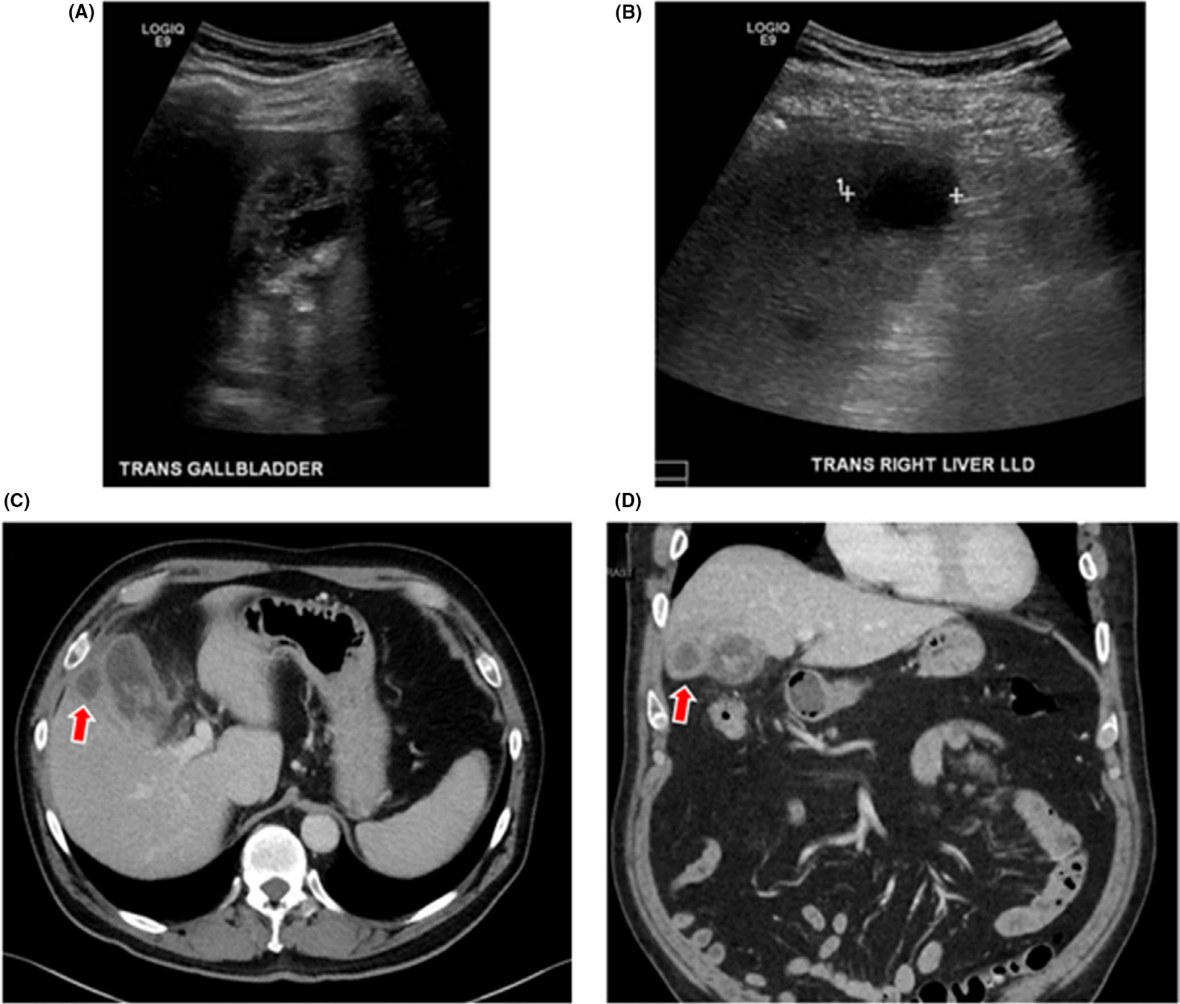
A 76‐year‐old man presented with minimal abdominal pain, nausea, emesis, and night sweats. Ultrasound showed cholelithiasis with thickened gall bladder (GB) walls (A) and 2.8 × 2.6 × 2 cm hypoechoic collection in the right hepatic lobe (B). Computed tomography scan showed thickened and irregular GB walls, pericholecystic stranding, and intraluminal membranes, suggestive of gangrenous cholecystitis (C,D). Fluid collection adjacent to GB (red arrows) is suggestive of pericholecystic abscess. The patient was successfully managed with percutaneous cholecystostomy

Percutaneous cholecystostomy may also have a role in milder presentations of AC. In patients with grade II AC, PC followed by LC has been shown to have better outcomes compared to EC, including lower rates of conversion to OC, less intraoperative bleeding, shorter duration of postoperative abdominal drainage, shorter hospital stays after cholecystectomy, lower incidence of respiratory failure, fewer admissions to the ICU, and, greater reversal of the pathologic process affecting the GB.^41^ There clearly is a subset of patients more optimally treated with PC (Figure 1).

Despite the benefits of PC, the decision to pursue PC should be carefully assessed on an individual basis. In a propensity‐matched cohort of Medicare patients with grade III AC, patients with PC for GB drainage had worse short‐ and long‐term outcomes compared to those without PC. Specifically, those with tube placement had significantly higher 30‐day, 90‐day, and 2‐year readmission and mortality rates compared to those without tube placement.^19^ They were also less likely to undergo cholecystectomy in the following 2 years after hospitalization, had longer hospital stays and more complications.^19^ One recent randomized trial from the Netherlands in patients with an APACHE II score of >7 was abandoned after they found significantly higher reintervention and morbidity in the PC group.^42^ These conflicting results imply that more specific studies need to be conducted on the precise indications for PC.

Despite conflicting data surrounding indications for PC, there is consensus that if the decision is made to pursue PC, it should be done early (<24 hours post‐symptom onset). For patients with inoperable severe AC, early PC has been shown to reduce length of hospital stay and procedure‐related bleeding when compared with PC performed after 24 hours.^43^

After PC, the optimal timing to remove the GB is controversial. One study that looked at early surgery (<72 hours) and delayed surgery (>72 hours) after PC reported higher incidence of postoperative complications and longer operation time for the early surgery group, although those with early surgery had shorter hospital stays.^44^ No difference in conversion rate between the two groups was observed. Another study reported early surgery after PC (<72 hours) had higher bleeding and longer operating times than delayed surgery (>5 days).^45^ Yet another reported no difference between early surgery (<10 days) and delayed surgery (>10 days) regarding complication rates, operating time, conversion rates, and total hospital stay.^46^ Thus, the precise timing of surgery after PC needs to be more thoroughly studied. Most surgeon in the USA will wait an interval of at least 4‐6 weeks.

## 6 SUMMARY AND CONCLUSION

Although AC is one of the most commonly treated surgical conditions, its diagnosis and management are complex and nuanced. There continue to be controversies surrounding its diagnosis and classification, the optimal approach based on the timing of operation in relation to the onset and magnitude of symptoms, and the appropriate use of PC. Operatively, LC can range from straightforward cases to some of the most challenging operations in abdominal surgery. Thus, any surgeon performing LC needs to be well‐versed in the various intra‐operative techniques and bail‐out options.

## DISCLOSURE

Conflicts of interest: the authors declare no conflicts of interest for this article.

## References

[1] Portincasa P , Moschetta A , Palasciano G . Cholesterol gallstone disease. Lancet. 2006;368:230–9. 16844493 10.1016/S0140-6736(06)69044-2

[2] Friedman GD . Natural history of asymptomatic and symptomatic gallstones. Am J Surg. 1993;165:399–404. 8480871 10.1016/s0002-9610(05)80930-4

[3] Gibney EJ . Asymptomatic gallstones. Br J Surg. 1990;77:368–72. 2187558 10.1002/bjs.1800770405

[4] Friedman GD , Raviola CA , Fireman B . Prognosis of gallstones with mild or no symptoms: 25 years of follow‐up in a health maintenance organization. J Clin Epidemiol. 1989;42:127–36. 2918322 10.1016/0895-4356(89)90086-3

[5] Ko CW , Lee SP . Gastrointestinal disorders of the critically ill. Biliary sludge and cholecystitis. Best Pract Res Clin Gastroenterol. 2003;17:383–96. 12763503 10.1016/s1521-6918(03)00026-x

[6] Indar AA , Beckingham IJ . Acute cholecystitis. BMJ. 2002;325:639–43. 12242178 10.1136/bmj.325.7365.639 PMC1124163

[7] Wiesen SM , Unger SW , Barkin JS , Edelman DS , Scott JS , Unger HM . Laparoscopic cholecystectomy: the procedure of choice for acute cholecystitis. Am J Gastroenterol. 1993;88:334–253. 8438837

[8] Zacks SL , Sandler RS , Rutledge R , Brown RS Jr . A population‐based cohort study comparing laparoscopic cholecystectomy and open cholecystectomy. Am J Gastroenterol. 2002;97:334–40. 11866270 10.1111/j.1572-0241.2002.05466.x

[9] Csikesz NG , Tseng JF , Shah SA . Trends in surgical management for acute cholecystitis. Surgery. 2008;144:283–9. 18656637 10.1016/j.surg.2008.03.033

[10] Navez B , Ungureanu F , Michiels M , et al. Surgical management of acute cholecystitis: results of a 2‐year prospective multicenter survey in Belgium. Surg Endosc. 2012;26:2436–45. 22407152 10.1007/s00464-012-2206-7

[11] Hirota M , Takada T , Kawarada Y , et al. Diagnostic criteria and severity assessment of acute cholecystitis: Tokyo Guidelines. J Hepatobiliary Pancreat Surg. 2007;14:78–82. 17252300 10.1007/s00534-006-1159-4 PMC2784516

[12] Yokoe M , Takada T , Mayumi T , et al. Accuracy of the Tokyo Guidelines for the diagnosis of acute cholangitis and cholecystitis taking into consideration the clinical practice pattern in Japan. J Hepatobiliary Pancreat Surg. 2011;18:250–253. 10.1007/s00534-010-0338-5 21042814

[13] Yokoe M , Takada T , Strasberg SM , et al. New diagnostic criteria and severity assessment of acute cholecystitis in revised Tokyo Guidelines. J Hepatobiliary Pancreat Surg. 2012;19:578–85. 10.1007/s00534-012-0548-0 PMC3429769 22872303

[14] Gonzalez‐Munoz JI , Franch‐Arcas G , Angoso‐Clavijo M , et al. Risk‐adjusted treatment selection and outcome of patients with acute cholecystitis. Langenbecks Arch Surg. 2017;402:607–14. 27704274 10.1007/s00423-016-1508-y

[15] Paul Wright G , Stilwell K , Johnson J , Hefty MT , Chung MH . Predicting length of stay and conversion to open cholecystectomy for acute cholecystitis using the 2013 Tokyo Guidelines in a US population. J Hepatobiliary Pancreat Surg. 2015;22:795–801. 10.1002/jhbp.284 26288122

[16] Yokoe M , Hata J , Takada T , et al. Tokyo Guidelines 2018: diagnostic criteria and severity grading of acute cholecystitis (with videos). J Hepatobiliary Pancreat Surg. 2018;25:41–54. 10.1002/jhbp.515 29032636

[17] Yokoe M , Takada T , Strasberg SM , et al. TG13 diagnostic criteria and severity grading of acute cholecystitis (with videos). J Hepatobiliary Pancreat Surg. 2013;20:35–46. 10.1007/s00534-012-0568-9 23340953

[18] Joseph B , Jehan F , Dacey M , et al. Evaluating the relevance of the 2013 Tokyo Guidelines for the diagnosis and management of cholecystitis. J Am Coll Surg. 2018;227(38–43):e1. 29580879 10.1016/j.jamcollsurg.2018.02.016

[19] Dimou FM , Adhikari D , Mehta HB , Riall TS . Outcomes in older patients with grade III cholecystitis and cholecystostomy tube placement: a propensity score analysis. J Am Coll Surg. 2017;224(502–11):e1. 28069529 10.1016/j.jamcollsurg.2016.12.021 PMC5367962

[20] Okamoto K , Suzuki K , Takada T , et al. Tokyo Guidelines 2018: flowchart for the management of acute cholecystitis. J Hepatobiliary Pancreat Surg. 2018;25:55–72. 10.1002/jhbp.516 29045062

[21] Kum CK , Eypasch E , Lefering R , Paul A , Neugebauer E , Troidl H . Laparoscopic cholecystectomy for acute cholecystitis: is it really safe? World J Surg. 1996;20:43–8; discussion 8‐9. 8588411 10.1007/s002689900008

[22] Kum CK , Goh PM , Isaac JR , Tekant Y , Ngoi SS . Laparoscopic cholecystectomy for acute cholecystitis. Br J Surg. 1994;81:1651–4. 7827896 10.1002/bjs.1800811130

[23] Shikata S , Noguchi Y , Fukui T . Early versus delayed cholecystectomy for acute cholecystitis: a meta‐analysis of randomized controlled trials. Surg Today. 2005;35:553–60. 15976952 10.1007/s00595-005-2998-3

[24] Cao AM , Eslick GD , Cox MR . Early laparoscopic cholecystectomy is superior to delayed acute cholecystitis: a meta‐analysis of case‐control studies. Surg Endosc. 2016;30:1172–82. 26139487 10.1007/s00464-015-4325-4

[25] Zafar SN , Obirieze A , Adesibikan B , Cornwell EE 3rd , Fullum TM , Tran DD . Optimal time for early laparoscopic cholecystectomy for acute cholecystitis. JAMA Surg. 2015;150:129–36. 25517723 10.1001/jamasurg.2014.2339

[26] Siddiqui T , MacDonald A , Chong PS , Jenkins JT . Early versus delayed laparoscopic cholecystectomy for acute cholecystitis: a meta‐analysis of randomized clinical trials. Am J Surg. 2008;195:40–253. 18070735 10.1016/j.amjsurg.2007.03.004

[27] Lau H , Lo CY , Patil NG , Yuen WK . Early versus delayed‐interval laparoscopic cholecystectomy for acute cholecystitis: a metaanalysis. Surg Endosc. 2006;20:82–253. 16247580 10.1007/s00464-005-0100-2

[28] Cao AM , Eslick GD , Cox MR . Early cholecystectomy is superior to delayed cholecystectomy for acute cholecystitis: a meta‐analysis. J Gastrointest Surg. 2015;19:848–57. 25749854 10.1007/s11605-015-2747-x

[29] To KB , Cherry‐Bukowiec JR , Englesbe MJ , et al. Emergent versus elective cholecystectomy: conversion rates and outcomes. Surg Infect. 2013;14:512–9. 10.1089/sur.2012.160 24274058

[30] Roulin D , Saadi A , Di Mare L , Demartines N , Halkic N . Early versus delayed cholecystectomy for acute cholecystitis, are the 72 hours still the rule?: a randomized trial. Ann Surg. 2016;264:717–22. 27741006 10.1097/SLA.0000000000001886

[31] Inoue K , Ueno T , Douchi D , et al. Risk factors for difficulty of laparoscopic cholecystectomy in grade II acute cholecystitis according to the Tokyo guidelines 2013. BMC Surg. 2017;17:114. 29183352 10.1186/s12893-017-0319-6 PMC5706415

[32] www.sages.org/safe‐cholecystectomy‐program/.

[33] Strasberg SM , Brunt LM . Rationale and use of the critical view of safety in laparoscopic cholecystectomy. J Am Coll Surg. 2010;211:132–8. 20610259 10.1016/j.jamcollsurg.2010.02.053

[34] Nijssen MA , Schreinemakers JM , Meyer Z , van der Schelling GP , Crolla RM , Rijken AM . Complications after laparoscopic cholecystectomy: a video evaluation study of whether the critical view of safety was reached. World J Surg. 2015;39:1798–803. 25711485 10.1007/s00268-015-2993-9

[35] Pesce A , Latteri S , Barchitta M , et al. Near‐infrared fluorescent cholangiography ‐ real‐time visualization of the biliary tree during elective laparoscopic cholecystectomy. HPB. 2018;20:538–45. 29292071 10.1016/j.hpb.2017.11.013

[36] Strasberg SM , Pucci MJ , Brunt LM , Deziel DJ . Subtotal cholecystectomy‐”fenestrating” vs “reconstituting” subtypes and the prevention of bile duct injury: definition of the optimal procedure in difficult operative conditions. J Am Coll Surg. 2016;222:89–96. 26521077 10.1016/j.jamcollsurg.2015.09.019

[37] Patterson EJ , McLoughlin RF , Mathieson JR , Cooperberg PL , MacFarlane JK . An alternative approach to acute cholecystitis. Percutaneous cholecystostomy and interval laparoscopic cholecystectomy. Surg Endosc. 1996;10:1185–8. 8939839 10.1007/s004649900275

[38] Bickel A , Hoffman RS , Loberant N , Weiss M , Eitan A . Timing of percutaneous cholecystostomy affects conversion rate of delayed laparoscopic cholecystectomy for severe acute cholecystitis. Surg Endosc. 2016;30:1028–33. 26139479 10.1007/s00464-015-4290-y

[39] Abi‐Haidar Y , Sanchez V , Williams SA , Itani KM . Revisiting percutaneous cholecystostomy for acute cholecystitis based on a 10‐year experience. Arch Surg. 2012;147:416–22. 22785633 10.1001/archsurg.2012.135

[40] Melloul E , Denys A , Demartines N , Calmes JM , Schafer M . Percutaneous drainage versus emergency cholecystectomy for the treatment of acute cholecystitis in critically ill patients: does it matter? World J Surg. 2011;35:826–33. 21318431 10.1007/s00268-011-0985-y

[41] Ke CW , Wu SD , Li YN . Emergency cholecystectomy versus percutaneous transhepatic gallbladder drainage followed by delayed cholecystectomy in patients with moderate acute cholecystitis. Zhonghua Yi Xue Za Zhi. 2018;98:768–72. 29562403 10.3760/cma.j.issn.0376-2491.2018.10.011

[42] Loozen CS , van Santvoort HC , van Duijvendijk P , et al. Laparoscopic cholecystectomy versus percutaneous catheter drainage for acute cholecystitis in high risk patients (CHOCOLATE): multicentre randomised clinical trial. BMJ. 2018;363:k3965. 30297544 10.1136/bmj.k3965 PMC6174331

[43] Chou CK , Lee KC , Chan CC , et al. Early percutaneous cholecystostomy in severe acute cholecystitis reduces the complication rate and duration of hospital stay. Medicine. 2015;94:e1096. 26166097 10.1097/MD.0000000000001096 PMC4504525

[44] Han IW , Jang JY , Kang MJ , Lee KB , Lee SE , Kim SW . Early versus delayed laparoscopic cholecystectomy after percutaneous transhepatic gallbladder drainage. J Hepatobiliary Pancreat Surg. 2012;19:187–93. 10.1007/s00534-011-0458-6 21938408

[45] Choi JW , Park SH , Choi SY , Kim HS , Kim TH . Comparison of clinical result between early laparoscopic cholecystectomy and delayed laparoscopic cholecystectomy after percutaneous transhepatic gallbladder drainage for patients with complicated acute cholecystitis. Korean J Hepatobiliary Pancreat Surg. 2012;16:147–53. 26388926 10.14701/kjhbps.2012.16.4.147 PMC4575000

[46] Jung WH , Park DE . Timing of cholecystectomy after percutaneous cholecystostomy for acute cholecystitis. Korean J Gastroenterol. 2015;66:209–14. 26493506 10.4166/kjg.2015.66.4.209

